# Postprandial glycemic response to a high-protein diabetes-specific nutritional shake compared to isocaloric instant oatmeal in people with type 2 diabetes: a randomized, controlled, crossover trial

**DOI:** 10.3389/fcdhc.2024.1399410

**Published:** 2024-06-05

**Authors:** Sara Thomas, Beth Besecker, Yong Choe, Elena Christofides

**Affiliations:** ^1^Scientific and Medical Affairs, Abbott Nutrition, Columbus, OH, United States; ^2^Endocrinology Research Associates (CRC), Columbus, OH, United States

**Keywords:** diabetes, postprandial glucose, GLP-1, nutrition, protein

## Abstract

**Introduction:**

Minimizing postprandial glucose response is an important goal for overall diabetes management. Diabetes-specific nutritional shakes (DSNS) have been clinically shown to minimize postprandial glucose response in people with type 2 diabetes (T2DM) compared to high-glycemic foods. However, it is unknown how a high-protein, low-fat DSNS impacts the GLP-1 response.

**Methods:**

We tested the postprandial glucose, insulin, and GLP-1 response to a high-protein, low-fat diabetes-specific nutritional shake (DSNS-HP) compared to isocaloric instant oatmeal (IOM) in a randomized, controlled, crossover study in adults with T2DM (n = 24). Participants were randomly selected to receive IOM or DSNS-HP on two test days. Glucose, insulin, and total GLP-1 concentration were measured at baseline and 15, 30, 45, 60, 90, 120, 180, and 240 min postprandially.

**Results:**

Compared to IOM, the glucose-positive area under the curve (pAUC) was significantly lower (*P* = .021). DSNS-HP significantly increased GLP-1 pAUC response by 213% (*P* <.001) with a corresponding increase in insulin pAUC (P = .033) compared to IOM.

**Discussion:**

A high-protein, low-fat DSNS leads to favorable changes in GLP-1 response and is a suitable option to minimize blood glucose response in people with type 2 diabetes.

## 1 Introduction

According to the 2022 National Diabetes Statistics Report, 130 million adults are living with diabetes or prediabetes, with 37.3 million people (approximately 1 in 10) diagnosed with diabetes (1). Diabetes can result in complications such as heart disease, stroke, blindness, kidney failure, and amputation of the legs and feet. One of the most important and recognized steps in diabetes management is the implementation of lifestyle recommendations that include improving diet and exercise along with behavioral strategies in conjunction with prescribed medications (2–6). Meal replacements can enhance dietary adherence via portion control and provide convenience while helping to meet nutritional needs and improve metabolic outcomes as part of lifestyle interventions (7, 8). Diabetes-specific nutritional shakes (DSNS) are meal replacements formulated with lower total carbohydrate and low glycemic profiles, including unsaturated fatty acids and moderate to high protein levels. Compared to high-glycemic carbohydrate sources, DSNS demonstrates improved postprandial glycemic response in people with T2DM (9–13). Modulation of postprandial glucose is important for overall glycemic control (14), so targeting the postprandial period by replacing high-glycemic carbohydrates with a DSNS could be an effective tool as part of lifestyle intervention for glycemic management in people with T2DM.

Glucagon-like peptide-1 (GLP-1) is an incretin hormone that plays a key role in appetite management and glucose homeostasis (15). In response to nutrient consumption, it is released from gastrointestinal cells, stimulates insulin secretion from pancreatic β-cells, inhibits the secretion of glucagon from α- cells, slows gastric emptying, and increases satiety. Previous studies showed that DSNS increased the release of GLP-1 while minimizing blood glucose response postprandially when compared to an isocaloric whole-food breakfast of oatmeal or a standard oral nutritional supplement in people with T2DM (9–11). This was attributed to the amount and type of carbohydrates and inclusion of long-chain fatty acids, such as monounsaturated fatty acids (MUFAs), in the study DSNS (9–11, 13, 16). Resistant maltodextrins and fermentable fiber undergo fermentation to produce short-chain fatty acids (SCFAs), which are shown to promote GLP-1 release (17). In addition, consumption of meals rich in MUFAs results in increased endogenous GLP-1 secretion in people with T2DM without significant effects on glucose or insulin (16, 18). Amino acids and oligopeptides from protein digestion also promote GLP-1 release (19, 20).

Historically, DSNS were formulated similarly, with a high MUFA content (>40% total energy), moderate carbohydrate (35%–40% total energy), and protein ≤ 15 g/serving. However, guidelines for diabetes management indicate that there is no ideal macronutrient ratio for people with diabetes, and different macronutrient profiles can be tailored to the person’s eating patterns, preferences, and metabolic goals (2). For example, people with T2DM are at higher risk for sarcopenia and may not be able to meet their protein recommendations (21, 22). Thus, a DSNS with higher protein can help add protein to the diet to maintain muscle in this population. However, it is unknown how a higher-protein, low-fat DSNS modulates glucose, insulin, and GLP-1 responses. Thus, this randomized controlled trial compared the impact of a high-protein, low-fat DSNS (DSNS-HP), which included low-glycemic carbohydrates (resistant maltodextrin and inulin), to isocaloric, instant oatmeal (IOM), a commonly consumed breakfast food, on postprandial glucose, insulin, and GLP-1 responses in people with T2DM.

## 2 Materials and methods

Using a randomized, controlled, two-arm crossover design at multiple study sites, we compared a serving size of DSNS-HP to an isocaloric amount of IOM on glycemic response. Adults (≥ 21 and ≤ 75 years) with T2DM (A1C level > 7% and ≤ 10%) managed by oral antihyperglycemic medications (excluding exogenous insulin, GLP-1 agonists, or DPP-4 inhibitors) were enrolled. Participants were required to meet the following additional enrollment criteria: BMI between > 18.5 and ≤ 40 kg/m^2^, men or non-pregnant, non-lactating women, willing to follow the protocol as described, stable dose of medications at least 2 months prior to screening, and at least a 2-week washout period between completion of a previous research study requiring ingestion of any study food or drug. We excluded participants with infection or disease (cardiovascular, renal, gastroparesis, hepatic, type 1 diabetes, cancer, gastrointestinal), inpatient surgery or receiving systemic corticosteroid treatment in the last 3 months, antibiotics in the last 3 weeks, active pregnancy or lactation, weight changes greater than 3 kg for 2 months prior to screening, eating disorder, severe dementia or delirium, history of significant neurological or psychiatric disorder, alcoholism or substance abuse, use of DSNS at more than one eating occasion per week in the last 3 months, and those taking any herbals, dietary supplements, or medications, other than the allowed anti-hyperglycemic medications, during the 4 weeks prior to the screening visit that might impact blood glucose. Capillary HbA1c (A1CNOW^®^+ System, pts Diagnostics, Whitestown, IN), medical history, demographic and baseline data, anthropometrics, and medications and dietary supplement use were collected at the screening visit. All participants gave their informed consent for inclusion before they participated in the study. The study was conducted in accordance with the Declaration of Helsinki; the protocol was approved by the institutional review board of Advarra (Columbia, MD, USA) (Pro00060214); and it was registered on ClinicalTrials.gov (NCT05154045).

Participants were randomized to receive DSNS-HP (Glucerna Protein Smart, 11-fl-oz, Abbott Nutrition, Columbus, OH) or isocaloric plain, instant oatmeal prepared with water according to the manufacturer’s instructions (The Quaker Oats Company, Chicago, IL) for a meal tolerance test (MTT) on two separate test days with a 4–14-day break between test days. Eligible participants were sequentially assigned a recruitment number in ascending numerical order. Randomization schedules were computer-generated using a pseudo-random permuted block algorithm. An electronic data capture system was utilized to assign participant numbers and randomize participants to treatments according to the generated randomization schedules. Participants were randomly allocated to intervention sequences so that they received the two treatments in balanced order. Participants were not blinded to the treatment allocation. Researchers accessing the study outcomes and labs that analyze the blood data were blinded to the treatment allocation. **Table 1** summarizes the nutritional composition of the test meals. As part of the MTT protocol, participants were required to fast for 8–14 h with no strenuous exercise or alcohol for 24 h prior. Participants completed a 3-day diet record to verify consumption of more than 150 g of carbohydrate per day and were asked to follow a similar meal pattern the day before each test day. A fasting capillary glucose measurement was collected (FreeStyle Precision Neo Blood Glucose Monitoring System, Abbott Diabetes Care, Inc., Alameda, CA) to confirm a fasting blood glucose < 180 mg/dL before the MTT could proceed. If the patient did not abide by the MTT protocol or their blood glucose was ≥180 mg/dL, they were allowed to reschedule one time per visit with a maximum of two reschedules during the whole study. Participants brought their morning dose of antihyperglycemic medications to the study site to take prior to the MTT. On each test day, blood was collected via venipuncture for 15 min (the baseline) before consuming the test meal. Participants consumed the test meal within 15 min, with the start of intake set at time point zero. Blood samples were collected at 15, 30, 45, 60, 90, 120, 180, and 240 min postprandially and analyzed for plasma glucose (enzymatic reference method with hexokinase, Cobas 6000 system C501, Roche Diagnostics, Indianapolis, IN, USA), serum insulin [Siemens Insulin (IRI)], two-site sandwich immunoassay using direct chemiluminescent technology (Advia Centaur XP, Siemens), and plasma GLP-1 (Total GLP-1 NL-ELISA, Mercodia, Worthington, OH, USA). Blood analyses were performed at Eurofins Central Laboratory, Lancaster, PA (insulin and glucose) and Mercodia AB, Uppsala, Sweden (GLP-1). Appetite and sensory data were collected for the DSNS-HP intervention only for exploratory purposes and are not reported here.

**Table 1:** Nutritional information per serving provided for the study product.

	DSNS-HP	Instant oatmeal + water
Serving size	330 mL	40 g + 237 mL water
Calories, kcal	150	150
Carbohydrates, g (% TE)	7 (11)	27 (72)
Dietary fiber, g	4	4.5
Fat, g (%TE)	1.5 (9)	3 (18)
Saturated fat, g (% TE)	0.5 (3)	0.75 (4)
Monounsaturated fat, g (% TE)	–	0.75 (4)
Polyunsaturated fat, g (% TE)	–	0.75 (4)
Protein, g (% TE)	30 (80)	6 (16)

DSNS**-**HP, diabetes-specific nutritional shake—high protein; TE, total energy.

A sample size estimation was made for the primary variable, the positive area under the curve for plasma glucose concentration over 0 to 240 min, to compare IOM and DSNS-HP. The assumptions for the estimation were made from a previously published study (5). A sample size of 20 participants, 10 per treatment sequence, had at least 80% power to detect an effect size of 1.36, which is twice the effect size (0.68) of the treatment difference, using a two-group t-test with a 0.05 two-sided significance level (SAS® Version 9.4 and SAS® Enterprise Guide Version 7.1). Based on an estimated 23% attrition rate, approximately 26 participants were targeted for enrollment to obtain 20 protocol-evaluable participants.

### 2.1 Statistical analyses

Each continuous variable was analyzed to compare the two treatment groups. If the sequence (carryover) effect was statistically significant, only period one data were analyzed using the Wilcoxon rank sum test (if non-normal) or analysis of variance (ANOVA). Otherwise, both periods were analyzed using the Wilcoxon rank sum test (if non-normal) or repeated measures ANOVA. All hypothesis tests, except tests for the sequence effect, were done using two-sided, 0.05-level tests. Tests for the sequence effect were done using two-sided, 0.10-level tests. The statistical analyses were completed using SAS® Version 9.4 and SAS® Enterprise Guide Version 8.3.

Planned postprandial collection times for glucose, insulin, and GLP-1 were at 15, 30, 45, 60, 90, 120, 180, and 240 min; ± 5 min for the first hour and ± 10 min for the subsequent hours. The measurements collected 15 min before consuming the test meal were used for the 0-min values. Values outside these planned postprandial measurement collection times were left missing. A small number (< 1%) of qualified missing values (≤ 3 missing at 0, 15, 30, 45, 60, 90, 120, 180, or 240 min) were imputed using the R function missForest (23, 24). Calculations for area under the curve from 0–240 min (AUC_0–240 min_), positive area under the curve from 0–240 min (pAUC_0–240 min_), peak value, adjusted peak value, peak time, and adjusted values were determined for glucose, insulin, and GLP-1. AUC_0–240 min_ was calculated by adding up the trapezoids defined by nine points (time, value) at 0, 15, 30, 45, 60, 90, 120, 180, and 240 min, and pAUC_0–240_ min was calculated by adding up only the portions of the AUC above the value at time zero. The peak value was the maximum value over 0–240 min, and the adjusted peak value was the peak value minus the value at time zero. Peak time was the first planned time to peak value during the time interval of 0–240 min. The adjusted value at individual time points was the value at the time point minus the value at time zero.

## 3 Results

### 3.1 Study participants

**Figure 1:**
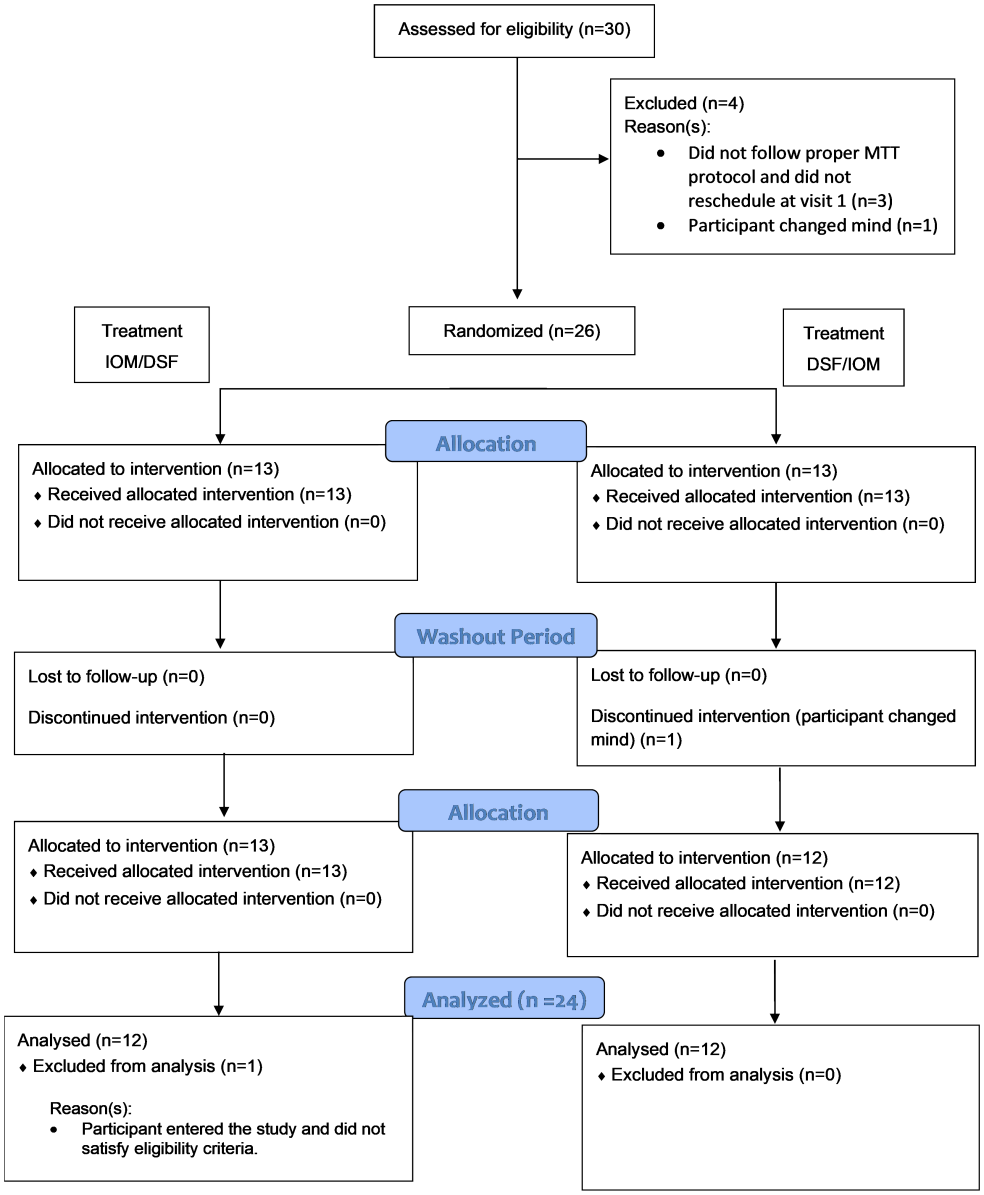
Consort flow diagram. MTT, meal tolerance test.

Out of the 30 participants assessed for eligibility, 26 were enrolled, and 24 were included in the evaluable data set (**Figure 1**). Two participants were excluded from the evaluable data set: one participant was enrolled but did not satisfy the eligibility criteria (the participant was using a DPP-4 inhibitor), and one participant changed their mind and did not appear for the second intervention visit. No safety concerns associated with the consumption of the test meals were identified. **Table 2** summarizes the demographic and baseline characteristics of protocol-evaluable participants.

**Table 2:** Protocol-evaluable participant demographic and baseline characteristics (mean ± SEM, n = 24).

Age (years)	61.6 ± 1.3
Sex, n (%) Male Female	* * 13 (54.2) 11 (45.8)
Hispanic/Latino, n (%) Not Hispanic/Latino, n (%) Unreported, n (%)	18 (75.0) 3 (12.5) 3 (12.5)
Race, n (%) African American White Asian	* * 4 (16.7) 19 (79.2) 1 (4.2)
Weight (kg)	92.12 ± 4.66
BMI	31.94 ± 1.11
Hemoglobin A1C (%)	7.67 ± 0.13
Duration of diabetes (years)	11.79 ± 1.25
Fasting blood glucose (mg/dL)	151 ± 6

### 3.2 Postprandial glucose response to DSNS-HP and IOM

**Figure 2:**
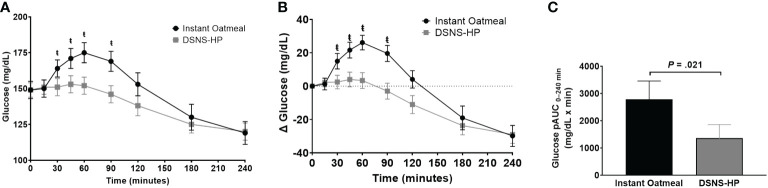
**(A)** Postprandial glucose response; **(B)** change from baseline postprandial glucose response; and **(C)** positive AUC_0–240_ min for glucose response following the consumption of instant oatmeal and DSNS-HP. Data are presented as mean ± SEM. ^ŧ^P <.05. pAUC, positive area under the curve; DSNS-HP, diabetes-specific nutritional shake—high protein.

Baseline glucose concentrations were not significantly different between IOM (LSM ± SE 151 ± 6 mg/dL) and DSNS-HP (LSM ± SE 151 ± 6 mg/dL) (*P* = .975). Overall, postprandial glucose response was lower after consuming DSNS-HP compared to IOM (**Figure 2**). Consumption of DSNS-HP led to an 80% reduction in median postprandial glucose pAUC_0–240 min_ [360 (IQR 0–1189)] compared to IOM [1768 (IQR 1225–3264)] (*P* = .021). Glucose and adjusted glucose concentrations were significantly lower for DSNS-HP at 30, 45, 60, and 90 min (*P* <.05). Adjusted peak glucose concentration was 57% lower after consuming DSNS-HP (LSM ± SE 14.2 ± 4.1 mg/dL) compared to IOM (LSM ± SE 32.9 ± 4.1 mg/dL) (*P* = .003). The median peak time for plasma glucose concentration occurred earlier for DSNS-HP [30 min (IQR 0–53)] compared to IOM [45 min (IQR 30–60)] (*P* = .026). Although adjusted postprandial blood glucose concentrations dropped below baseline levels for DSNS-HP between 60 and 120 min, blood glucose concentrations remained above 100 mg/dL throughout the study.

### 3.3 Postprandial insulin response to DSNS-HP and IOM

**Figure 3:**
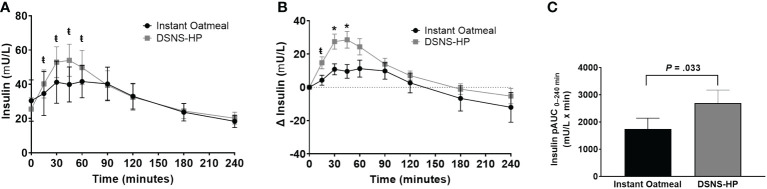
**(A)** Postprandial insulin response; **(B)** change from baseline postprandial insulin response; and **(C)** positive AUC_0_**_–_**_240_ min for insulin response following the consumption of instant oatmeal and DSNS-HP. Data are presented as mean ± SEM. ^*^P <.001, ^ŧ^P <.05. pAUC, positive area under the curve; DSNS-HP, diabetes-specific nutritional shake—high protein.

Baseline insulin concentrations were not significantly different between the two treatments. Compared to IOM, pAUC_0–240 min_ for insulin response was significantly higher after consuming DSNS-HP (*P* = .033; **Figure 3**). Similarly, insulin and adjusted insulin concentrations were significantly higher at time points 15–60 min and 15–45 min, respectively, for DSNS-HP compared to IOM (all *P* <.05). Median peak insulin and adjusted peak insulin concentrations were significantly higher for DSNS-HP [50.6 mU/L (IQR 28.2–74.8); 27.1 mU/L (IQR 13.5–48.4)] compared to IOM [33.9 mU/L (IQR 22.3–59.9); 14.6 mU/L (IQR 8.4–30.4)] (*P* = .021; *P* = .014). No significant difference was found in time to peak insulin concentration between the two treatments (*P* = .281). *Post hoc* analysis of early-phase insulin response (pAUC from 0–30 min) showed an increase in median from pAUC_0–30 min_ for DSNS-HP [274 (IQR 133–643)] compared to IOM [82 (IQR 8–222)] (*P* = .001).

### 3.4 Postprandial GLP-1 response to DSNS-HP and IOM

**Figure 4:**
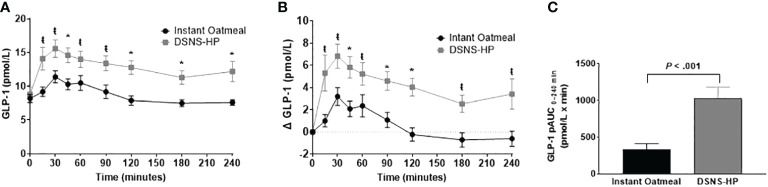
**(A)** Postprandial GLP-1 response; **(B)** change from baseline postprandial GLP-1 response; and **(C)** positive AUC_0_**_–_**_240_ min for GLP-1 response following the consumption of instant oatmeal and DSNS-HP. Data are presented as mean ± SEM. ^*^P <.001, ^ŧ^P <.05. pAUC, positive area under the curve; DSNS-HP, diabetes-specific nutritional shake—high protein; and GLP-1, glucagon-like peptide-1.

Baseline GLP-1 concentrations were comparable between DSNS-HP and IOM (**Figure 4**). Consumption of DSNS-HP resulted in an overall higher GLP-1 response compared to IOM. Positive AUC_0–240 min_ for postprandial GLP-1 response increased by 213% for DSNS-HP (LSM ± SE 1012 ± 121) compared to IOM (LSM ± SE 323 ± 121) (*P* <.001). Compared to IOM, GLP-1 and adjusted GLP-1 concentrations were significantly higher at all time points (15–240 min) after consuming DSNS-HP. Median adjusted peak GLP-1 concentration was 200% higher for DSNS-HP [9.0 pmol/L (IQR 4.7–11.1)] compared to IOM [3.0 pmol/L (IQR 1.5–7.3)] (*P* = .002). However, the time to peak GLP-1 concentration was not significantly different between the two treatments (*P* = 0.19).

## 4 Discussion

This randomized, crossover clinical trial showed that compared to isocaloric IOM, a high-protein, low-fat DSNS containing low-glycemic carbohydrates reduced postprandial glycemic response while increasing GLP-1 and insulin response in people with type 2 diabetes. These data are mostly consistent with prior DSNS glucose response studies, but with some distinct differences highlighted here (9–11, 13).

As hypothesized, the primary variable, pAUC for glucose response, was significantly lower for DSNS-HP compared to an isocaloric breakfast of IOM. This is consistent with other studies comparing isocaloric DSNS to oatmeal or a standard oral nutritional supplement and is attributed to the lower amount and type of carbohydrate in the DSNS (9–11, 13, 25). Compared to previous DSNS studied, DSNS-HP is lower in carbohydrates (7 g vs. 16–29 g), but similarly contains low glycemic carbohydrate sources. DSNS-HP contains resistant maltodextrin and inulin, both of which result in a lower glycemic response when used to replace high-glycemic carbohydrates in foods (26, 27). Although participants consuming both IOM and DSNS-HP had equivalent baseline glucose levels (~150 mg/dL), DSNS-HP glucose concentration peaked earlier (30 vs. 45 min) before dropping below baseline levels after 60 min, whereas glucose concentration for IOM continued to rise before going below baseline levels after 120 min. Although both IOM and DSNS-HP glucose concentrations dropped below baseline levels, blood glucose levels remained above 100 mg/dL throughout the postprandial period.

The increase in GLP-1 after eating is not unexpected and is a normal glucose-dependent response related to nutrient detection, specifically glucose, in the intestinal L-cells and gastric distention based on the volume of food in the stomach (28). However, the oatmeal, higher in carbohydrate and volume, failed to increase the GLP-1 concentration equivalent to DSNS-HP. Data from previous studies also showed a similar significant increase in GLP-1 response to DSNS compared to IOM, which was attributed to the low-glycemic carbohydrate blends and MUFA-rich formulations (9, 11). However, DSNS-HP is lower in fat and carbohydrate and higher in protein, indicating a different mechanism of action. Like glucose and fatty acids, amino acids and oligopeptides from protein digestion may stimulate GLP-1 release through a similar mechanism (29). A Meta-analysis found a significant increase in GLP-1 following acute protein dose (<5.5 h) interventions. A subgroup analysis of these data showed that ≥ 35 g of protein drove this increase (30). Though DSNS-HP contains less than this amount of protein (30 g/serving), it additionally contains fermentable fibers, which may promote GLP-1 via the production of short-chain fatty acids (29). Therefore, the combined action of high protein and fermentable fiber could explain the increase in GLP-1 response to DSNS-HP compared to IOM.

Interestingly, DSNS-HP showed a robust increase in plasma GLP-1 concentrations at all time points and remained elevated above baseline at 240 min, which has potential implications for the subsequent meal. Called the second-meal phenomenon, oral glucose tolerance is improved after a previously consumed glucose load (31, 32). This phenomenon also occurs after the consumption of mixed-macronutrient-containing meals and is preserved in T2DM (32). Efficacy is dependent on the nutrient composition of the prior meal. Meals higher in protein and low glycemic index carbohydrates show altered incretin responses and lower glycemic responses to a second meal (33, 34). Minimizations of glycemic response, including gastric emptying, early-phase insulin secretion, hepatic glucose output, and muscle glucose uptake, are potential mechanisms (35). Since people with T2DM have an impaired GLP-1 response compared to healthy individuals, its restoration could improve the early-phase insulin response (28, 36–38). DSNS containing nutrients that promote these mechanisms could potentially impact the second meal. Only one study investigated the impact of a DSNS on subsequent meals. This randomized pilot study in people with T2DM showed that replacing a usual breakfast and an afternoon snack with a DSNS containing 15 g protein resulted in greater reductions in glucose response compared to a habitual self-selected diet (12). However, no second meal effect on glycemic response was noted for this DSNS and the self-selected diet. Although the DSNS contained low glycemic index carbohydrates, it is possible that 15 g of protein was not enough to modify the incretin response to impact the second meal. Further studies are needed to determine if the second meal response can be manipulated by DSNS with differing macronutrient content, especially those with higher protein.

In addition to increasing GLP-1 response, DSNS-HP resulted in a subsequent increase in insulin response compared to IOM. Endogenous GLP-1 is released from the L-cells of the gastrointestinal tract in response to food to induce glucose-dependent insulin secretion (29). Adjusted GLP-1 concentrations were highest between time points 15 min and 60 min, which corresponds with the significantly higher adjusted insulin concentrations between time points 15 min and 45 min and the significant increase in the early-phase insulin response reflected by the increase in median pAUC_0–30 min_ for DSNS-HP compared to IOM. This suggests that the increase in GLP-1 response to DSNS-HP is directly involved in increasing the early-phase insulin response. This is important since early-phase insulin response is lost in patients with impaired glucose tolerance or early-stage T2DM and is inversely associated with 2-h glucose concentrations (39). Protein can also directly stimulate insulin secretion in the presence of carbohydrates, particularly in the early phase, with little impact on the glycemic response (19).

Two previous studies compared postprandial incretin responses to oatmeal and DSNS in people with T2DM, with varied results for insulin response. One study compared isocaloric instant oatmeal (200 kcals, 8 g protein, 38 g carbohydrate, and 4 g fat) to a MUFA-rich DSNS (216 kcals, 10 g protein, 29 g carbohydrate, and 8.1 g fat), and the data showed a significantly higher pAUC for insulin response and a higher peak and adjusted peak insulin concentration for the DSNS compared to instant oatmeal over 180 min (9). Like DSNS-HP, the higher insulin response to the study DSNS corresponded with a significantly higher GLP-1 response and minimized blood glucose response. In contrast, a second study compared a similar DSNS formulation (200 kcals, 10 g protein, 26 g carbohydrate, and 7 g fat) and a higher protein DSNS (200 kcals, 15 g protein, 27 g carbohydrate, and 7 g fat) to isocaloric oatmeal (11). The data showed no significant increase in insulin pAUC over 240 min between any of the treatments, despite a higher pAUC_0–240 min_ for the GLP-1 response and a lower pAUC_0–240 min_ for the glucose response to both DSNS compared to IOM. However, the 15-g protein DSNS showed a significantly higher insulin pAUC_0–120 min_ compared to IOM. This difference was attributed to the higher amount of protein and branched-chain amino acid composition, specifically insulinotropic leucine and phenylalanine. A higher protein amount along with low glycemic carbohydrates and fermentable fiber may also explain the reduced postprandial glucose pAUC_0–240 min_ response to DSNS-HP and concurrently higher GLP-1 and insulin pAUC_0–240 min_ responses. The overall higher insulin response to DSNS-HP appears to be driven by the robust early-phase insulin response, which resulted in a higher insulin pAUC_0–30 min_ compared to IOM. This is likely induced by the 30 g of protein through direct stimulation of insulin release or indirectly through GLP-1 secretion.

These were acute studies, which is one of the limitations of extrapolating the benefits to long-term metabolic outcomes. However, minimizing postprandial glucose response is an important target for long-term glycemic management to lower the risk of diabetes complications. While the interventions within each study were isocaloric, differences in volume could impact study outcomes. Participants were not blinded to the treatment options due to the inherent differences in IOM and DSNS-HP. Other measures impacting the glycemic response, such as glucagon and glucose-dependent insulinotropic peptide (GIP), were not measured. In addition, the sample size used in this study was small, so additional studies are warranted to see if the results are replicated in other populations. Although subjective appetite was evaluated as an exploratory outcome for DSNS-HP, it was not compared to IOM, and thus the results are not reported here. Future studies should consider comparing postprandial subjective satiety, satiety hormones and the rate of gastric emptying, which are both impacted by GLP-1. Studies comparing the impact of DSNS with different macronutrient profiles on glycemic response to breakfast and subsequent meals are warranted to better personalize medical nutrition therapy for people with diabetes.

## 5 Conclusions

The results of this study show that a high-protein, low-fat DSNS minimizes glycemic response while promoting increased GLP-1 and early-phase insulin response compared to IOM. This study extends the existing scientific evidence supporting the use of DSNS in T2DM and is the first study, to our knowledge, that provides data on the GLP-1 response to a high-protein, low-fat DSNS where protein appears to be the main driver of GLP-1 and insulin response. Thus, a high-protein, low-fat DSNS is a suitable option to incorporate into diabetes management to meet the nutritional needs of people with T2DM.

## Data availability statement

The raw data supporting the conclusions of this article will be made available by the authors, without undue reservation.

## Ethics statement

The studies involving humans were approved by Advarra Institutional Review Board. The studies were conducted in accordance with the local legislation and institutional requirements. The participants provided their written informed consent to participate in this study.

## Author contributions

ST: Conceptualization, Investigation, Visualization, Writing – original draft, Writing – review & editing. BB: Writing – review & editing. YC: Conceptualization, Data curation, Formal analysis, Methodology, Validation, Writing – review & editing. EC: Writing – review & editing.
